# Local dimension-reduced dynamical spatio-temporal models for resting state network estimation

**DOI:** 10.1007/s40708-015-0011-5

**Published:** 2015-02-03

**Authors:** Gilson Vieira, Edson Amaro, Luiz A. Baccalá

**Affiliations:** 1 Inter-institutional Grad Program on Bioinformatics, University of São Paulo, São Paulo, Brazil; 2 LIM-44, Department of Radiology, Faculty of Medicine, University of São Paulo, São Paulo, Brazil; 3 Escola Politécnica, University of São Paulo, São Paulo, Brazil

**Keywords:** Resting state fMRI, Dynamical spatio-temporal models, Brain connectivity, Sparsity

## Abstract

To overcome the limitations of independent component analysis (ICA), today’s most popular analysis tool for investigating whole-brain spatial activation in resting state functional magnetic resonance imaging (fMRI), we present a new class of local dimension-reduced dynamical spatio-temporal model which dispenses the independence assumptions that severely limit deeper connectivity descriptions between spatial components. The new method combines novel concepts of group sparsity with contiguity-constrained clusterization to produce physiologically consistent regions of interest in illustrative fMRI data whose causal interactions may then be easily estimated, something impossible under the usual ICA assumptions.

## Introduction

There is an ever-growing and pressing need for accurately describing how brain regions are dynamically interrelated in resting state fMRI [4]. Thanks to the nature of BOLD signals, resting state interactions cannot be split into separate space and time descriptions, especially if the focus lies on characterizing spatial changes associated to a small number of regions of interest. The chief challenge is that any dynamical spatio-temporal model (DSTM) of fMRI datasets demands many parameters to describe what is also a large number of observed variables which, nonetheless, enjoy a great deal of spatial redundancy [3, 5, 37]. Estimating the spatial origin of signal variability for only relatively short sample sizes using DSTMs is problematic especially under the rather usual unfavourable signal-to-noise ratio (SNR) conditions [8, 24, 28, 34].

To circumvent limitations of modelling high-dimensionality systems, Wikle and Cressie [33] proposed dimension-reduced DSTMs aimed at capturing nonstationary spatial dependence under optimal state representations using Kalman filtering. In their formulation of DSTM, they invoke an a priori defined orthogonal basis to expand the redistribution kernel of a discrete time/continuous space, linear integro-difference equation (IDE) in terms of a finite linear combination of spatial components [33]. This idea was further supported in [14] and extended in [26] who considered parametrized redistribution kernels of arbitrary shape that meet homogeneity conditions in both space and time. Even though the base changes of [33] improve the understanding of high-dimensional processes, they by no means ensure sparse solutions which are key to achieving statistically robust dynamical descriptions.

Model robustness has alternatively been sought by indirect means as, for example, thru LASSO regression [29] and basis pursuit [6] for model selection and denoising, or sparse component analysis for blind source separation [39] and finally by iterative thresholding algorithms for image deconvolution and reconstruction [12, 17]. The latter methods seek sparsity by maximizing a penalized loss function in a compromise between the goodness of fit and the number of basis elements that make up the signal. Recently, more attention has been given to group sparsity, where groups of variables are selected/shrunken simultaneously rather than individually (for a review see [2]). This is achieved by minimizing an objective function that includes a quadratic error term added to a regularization term that considers a priori beliefs or data-driven analysis to induce group sparsity [35, 36, 38].

The present paper extends the results in [31] about local dimension-reduced DSTMs (LDSTMs) involving state-space formulations that are suited to datasets of high dimensionality such as fMRI. LDSTMs take advantage of a sparsifying spatial wavelet transformation to represent the data thru fewer significant parameters which are then combined via sparsity and contiguity-constrained clustering to initialize the observation matrix and sources of a tailored expectation maximization (EM) algorithm. The main assumptions here are that the system is overdetermined (there exist more observed signals than sources) and that the columns of the observation matrix act as point-spreading functions (see Sect. 2). Finally, results are gauged using simulated data (Sect. 4) followed by further method illustration with directed connectivity disclosure using real fMRI resting state data.

## Problem formulation

**Fig. 1:**
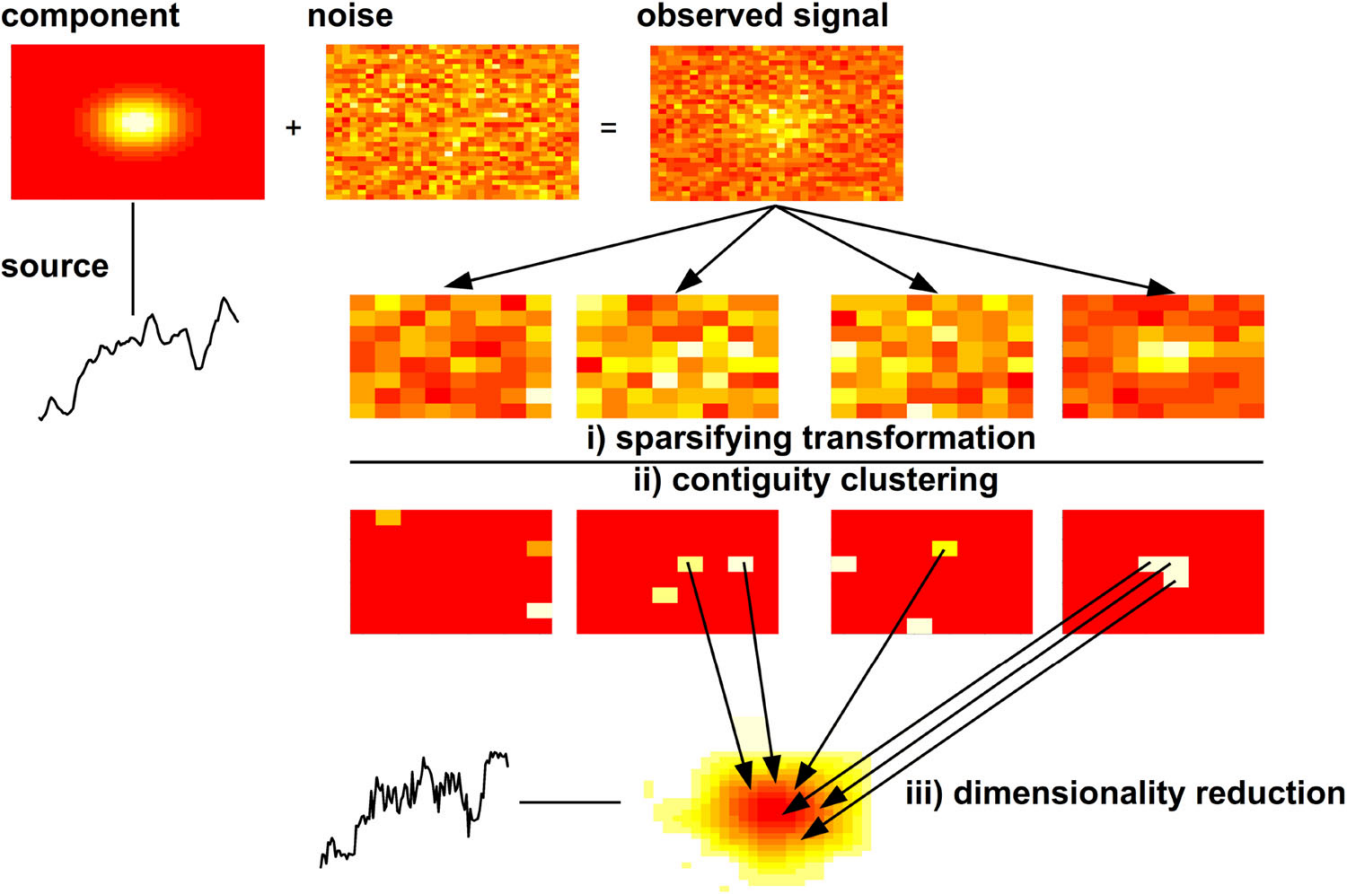
The main algorithm consists of (*i*) the application of a sparsifying spatial wavelet transformation, resulting into a description in terms of wavelet coefficient time series, (*ii*) contiguity-constrained clustering of the time series of wavelet coefficients by grouping only nearby coefficients and (*iii*) estimation of the observation matrix and system states by linear dimensionality reduction of the identified clusters

DSTM problems may be formulated as state-space models (see [9] for a comprehensive review of DSTM) where space-related measurements ${{z}}_{t}$ depend on the dynamical evolution of a suitably defined source vector ${{x}}_{t}$ through a linear gaussian model

1 $$\begin{aligned} {\mathbf{{x}}}_{t}&= \mathbf{\sum \nolimits }_{l=1}^{L} \mathbf{{{H}}}_{l} \mathbf{{{x}}}_{t-l}+\mathbf{{{w}}}_{t} \end{aligned}$$

2 $$\begin{aligned} \mathbf{{{z}}}_{t}&= \mathbf{{{A}} {{x}}}_{t} + \mathbf{{{v}}}_{t}, \end{aligned}$$

where $$\mathbf{{{z}}}_{t}$$ is an $$M$$ dimensional column vector of observed signals at time $$t, \mathbf{{{x}}}_{t}$$ is an $$K$$ dimensional column vector of unknown source vectors, $$\mathbf{{{A}}}$$ is an unknown $$M \times K$$ observation matrix, $$\mathbf{{{H}}}_{l}$$ for $$1 \le l \le L$$ are unknown $$K \times K$$ matrices that describe source vector dynamics, $$\mathbf{{{w}}}_{t}$$ is an innovation process and $$\mathbf{{{v}}}_{t}$$ is an additive noise. Both $$\mathbf{{{w}}}_{t} $$ and $$\mathbf{{{v}}}_{t}$$ are assumed zero mean gaussian, respectively, with covariance $$\mathbf{{{Q}}}$$ and $$\mathbf{{{R}}}$$. The $$\mathbf{{{H}}}_{l}$$ matrices, the observation matrix $$\mathbf{{{A}}}$$ together with $$\mathbf{{{Q}}}$$ and $$\mathbf{{{R}}}$$ and $$\mathbf{{{x}}}_{t}$$ must be inferred from $${{z}}_{t} $$. For added generality, Eq. (1) is presented in a slightly extended form compared to the corresponding model in [31].

Under the latter premises, the log-likelihood of model (1, 2) is given by

3 $$\begin{aligned} \log \,p \big (\mathbf{{{x}}},\mathbf{{{A}}},\mathbf{{{R}}},\mathbf{{{H}}}_{1},\ldots ,\mathbf{{{H}}}_{L},\mathbf{{{Q}}}|\mathbf{{{z}}}\big ) &= -\frac{T}{2}\log {|\mathbf {{{R}}}|}-\frac{1}{2}\sum _{t=1}^{T}({\mathbf{{z}}}_{t} - \mathbf{{{A}} {{x}}}_{t})^{{T}} \mathbf{{{R}}}^{-1} ({\mathbf{z}}_{t} - \mathbf {{{A}} {{x}}}_{t}) \nonumber \\ &\quad -\frac{T-1}{2}\log {|\mathbf {{{Q}}}|}-\frac{1}{2}\sum _{t=L+1}^{T}\left( {\mathbf{x}}_{t} - \sum _{l=1}^{L}\mathbf {{{H}}}_{l} \mathbf {{{x}}}_{t-l}\right) ^{{T}} \mathbf {{{Q}}}^{-1} \left( \mathbf {{{x}}}_{t} - \sum _{l=1}^{L} {\mathbf{H}}_{l} {{x}}_{t-l}\right) , \end{aligned}$$

where ${{z}} = {\mathrm {vec}}(\left[ {\mathbf {{z}}_{1}} \cdots {\mathbf {{z}}_{T}} \right] ), {\mathbf{x}} = {\mathrm {vec}}(\left[ {\mathbf {x}}_{1} \cdots {\mathbf {x}}_{T} \right] )$ and ${\mathrm {vec}}$ stands for the column stacking operator [27].

The EM algorithm has long been the favourite tool to solve (1,2) for ${\mathbf {x}}_{t}$ because (3) is sure to converge to at least a local maximum [13, 27]. The traditional EM algorithm starts with randomly generated solutions for all parameters and then proceeds by re-iterating its two main steps until the maximum of (3) is attained. It begins with the E-step where the unknown ${\mathbf {x}}_{t}$ are replaced by their expected values given the data and current model parameter estimates. Under gaussian assumptions, the expected system ${\mathbf {x}}_{t}$ are obtained via the Rauch–Tung–Striebel (RTS) smoother [25]. In the second algorithm step, the M-step, one estimates model parameters by maximizing the conditional expected likelihood from the previous E-step. In practice, EM algorithm performance degrades rapidly for high-dimensional systems under (1,2). Its solution may even become indeterminate and improper initialization, in fact, often deteriorates estimate quality.

To achieve robust EM solutions, we take into account two common neuroscientist concerns as to what constitute meaningful brain activity components: (a) ${\mathbf {x}}_{t}$ be an economic (i.e. compact/low dimensional) dynamical representation of the brain resting state fMRI dataset as a whole and (b) solutions must be spatially localized, i.e. their associated activation areas mathematically reflect point-spreading functions. We show that the latter assumptions not only allows estimating (1,2) parameters but also ${\mathbf {x}}_{t}$ using the simpler Local Sparse Component Analysis discussed in [32] on ${\mathbf {z}}_{t}$. The nutshell description of the present algorithm is represented in Fig. 1. The aim is to find initial estimators for the observation matrix and system states which are used to initialize a EM algorithm for maximization of (3).

## Algorithm details

### Sparsifying spatial wavelet transformation

Given $\{\phi _{m}\}_{1 \,\le m \,\le M}$ an wavelet basis in ${\mathbf {R}}^M$, the first step is to calculate the wavelet representation of the matrix of observations ${\mathbf {Z}} \equiv (z_{m,t})_{m,t}$ for $1 \le m \le M$ and $1 \le t \le T$

4 $$\begin{aligned} \hat{\mathbf {Z}} \equiv (\hat{z}_{m,t})_{m,t} = (\langle {\mathbf {z}}_{t}, \phi _{m} \rangle )_{m,t} = \varvec{\varPhi }{\mathbf {Z}}, \end{aligned}$$

where $\varvec{\varPhi }$ is the $M \times M$ orthonormal matrix, whose rows are the $\phi _{m}$’s. With obvious notation, ${\mathbf {Z}} = {\mathbf {S}} + {\mathbf {V}}$, where ${\mathbf {S}} = {\mathbf {A}} {\mathbf {X}}$, and $\hat{\mathbf {Z}} = \hat{\mathbf {S}} + \hat{{\mathbf {V}}}$. The transform $\varvec{\varPhi }$ should be chosen such that a tailored clustering of the rows of $\hat{\mathbf {S}}$ provides the elements that approximate the rows of ${\mathbf {X}}$. But before this step, $\hat{\mathbf {S}}$ must be estimated using the sparsity assumption which implies finding a sparse representation of $\hat{\mathbf {Z}}$ that captures its intrinsic degrees of freedom.

By considering that ${\mathbf {s}}_{t} = {\mathbf {A}} {\mathbf {x}}_{t}$ admits a sparse representation lying in ${\mathbf {B}}^{s}_{1,1}$, a particular kind of Besov space [23], approximating ${\mathbf {z}}_{t}$ by ${\mathbf {s}}_{t} \in {\mathbf {B}}^{s}_{1,1},$ can be expressed by adding a penalization term to $\Vert {\mathbf {z}}_{t} - {\mathbf {s}}_{t}\Vert _{2}^{2}$ requiring that $\Vert {\mathbf {s}}_{t} \Vert _{s,1}$ be small, where $\Vert {\mathbf {s}}_{t} \Vert _{s,1}$ is the ${\mathbf {B}}^{s}_{1,1}$ norm of ${\mathbf {s}}_{t}$. In other words, we want to minimize the following function:

5 $$\begin{aligned} f({\mathbf {s}}_{t}) = \Vert {\mathbf {z}}_{t} - {\mathbf {s}}_{t}\Vert _{2}^{2} + \Vert {\mathbf {s}}_{t} \Vert _{s,1} = \Vert {\mathbf {z}}_{t} - {\mathbf {s}}_{t}\Vert _{2}^{2} + \sum _{m} { \lambda }_{m} |{\hat{ s}}_{m,t}|, \end{aligned}$$

where ${\hat{ s}}_{m,t} = \langle {\mathbf {s}}_{t}, \phi _{m} \rangle$ and ${ \lambda }_{m} > 0$ for $1\le m \le M$ are regularization parameters [12].

For each $t$, the above function is coercive and strictly convex which means that it has a unique global minimum. If ${ \lambda }_{m} = \lambda,$ the minimum value of (5) is obtained via the soft-thresholding operator [15]

6 $$\begin{aligned} \hat{ s}_{m,t} = {\mathrm {sign}} ({\hat{ z}}_{m,t})\max (|{\hat{ z}}_{m,t}|-\lambda ,0). \end{aligned}$$

Since $\hat{ s}_{m,t}$ can be zero for some values of $t$ but not for others, the estimator (6) does not ensure sparsity of ${{ s}}_{t}$ over time even for large $\lambda$ values. To overcome this problem, we propose tying $\hat{ s}_{m,t}$ for $1\le t \le T$ together and using a recently introduced group-separable regularizer for the functional (5) but in the wavelet domain

7 $$\begin{aligned} \min _{\hat{\mathbf {s}}^{m}} \frac{1}{2} \Vert \hat{\mathbf {z}}^{m} - \hat{\mathbf {s}}^{m}\Vert _{2}^{2} + \lambda _{m} \Vert \hat{\mathbf {s}}^{m}\Vert _{2}, \end{aligned}$$

where $\hat{\mathbf {z}}^{m}$ and $\hat{\mathbf {s}}^{m}$ are the $m$-th rows of $\hat{\mathbf {Z}}$ and $\hat{\mathbf {S}},$ respectively. Given $\lambda _{m}$, solving (7) is achieved by the vector soft-thresholding operator [7, 35]

8 $$\begin{aligned} \hat{\mathbf {s}}^{m} = \frac{\max \left( \Vert \hat{\mathbf {z}}^{m}\Vert _{2} - \lambda _{m},0\right) }{\Vert \hat{\mathbf {z}}^{m}\Vert _{2}} \hat{\mathbf {z}}^{m}. \end{aligned}$$

In practice, we still need to estimate $\lambda _{m}$ in (8) for signal denoising. Since $\varvec{\varPhi }$ is orthogonal, if ${\mathbf {R}} = {\upsigma }^{2}{\mathbf {I}}_{M\times M}$, then ${\hat{{\mathbf {v}} }}^{m} \sim {\mathcal {N}}\left( {\mathbf {0}},{\upsigma }^{2}{\mathbf {I}}_{T\times T}\right)$, where ${\hat{{\mathbf {v}} }}^{m}$ is the $m$-th row of $\hat{{\mathbf {V}}}$. For very large datasets, this assumption is quite strong but commonly employed in literature. As ${\mathbf {z}}_{t}$ is sparse under $\varvec{\varPhi }$, most of $\{ \hat{s}_{m,t} \}_{\forall m}$ must be zero. Provided that fifty percent of $\{ \hat{s}_{m,t}\}_{\forall m}$ are zero, the following unbiased estimator for ${\upsigma }^{2}$ can be defined

9 $$\begin{aligned} \hat{\upsigma }^{2} = \hbox {median}_{\forall m} \hat{\hbox {VAR}} \{ \hat{z}_{m,t} \}, \end{aligned}$$

where $\hat{\hbox {VAR}}$ denotes temporal sample covariance.

If ${\hbox {VAR} }\{ \hat{s}_{m,t} \} = 0$, we have that $\hat{z}_{m,t}$ are i.i.d normal variables, so

10 $$\begin{aligned} \frac{(N-1) {\hat{\hbox {VAR}}} \{\hat{z}_{m,t}\}}{\upsigma ^{2}} \sim \chi _{N-1}^{2} \end{aligned}$$

implies that an interval with $(1-\alpha )$ confidence for ${\upsigma ^{2}}$ is given by

11 $$\begin{aligned} \Bigg [\frac{(N-1){{\hat{\upsigma }^{2}}} }{\chi _{1-\alpha /2,N-1}^{2}},\frac{(N-1){{\hat{\upsigma }^{2}}} }{\chi _{\alpha /2,N-1}^{2}}\Bigg ], \end{aligned}$$

where $\chi _{\xi ,\nu }^{2}$ is the $\xi$-th percentile of the chi-square distribution with $\nu$ degrees of freedom. Since $\Vert \hat{\mathbf {z}}^{m}\Vert _{2} = (N-1) \hat{\hbox {VAR}} \{\hat{z}_{m,t}\}$, (11) leads to $\lambda _{ m}$ given by

12 $$\begin{aligned} \lambda _{ m} = \frac{(N-1)^{2}{{\hat{\upsigma }^{2}}} }{\chi _{\alpha /2,N-1}^{2}}, \end{aligned}$$

with $$\alpha = 0.05/M$$.

### Contiguity-constrained clustering

The next step consists of determining which time series of wavelet coefficients $\hat{\mathbf {s}}^{m}$ are associated to each spatial component ${\mathbf {a}}_{k} {\mathbf {x}}^{k}$, where ${\mathbf {a}}_{k}$ is the $k$-th column of ${\mathbf {A}}$ and ${\mathbf {x}}^{k}$ is the $k$-th row of ${\mathbf {X}}$. For this, we use the spatial localization assumption. As the columns of the observation matrix are point-spreading functions, they should be perfectly described by wavelet coefficients forming localized spatial patterns. In this case, each spatial component can be determined using a clustering algorithm enforcing spatial contiguity. One way of achieving this is to apply complete linkage hierarchical clustering with the help of a dissimilarity measure that combines the time series temporal correlation and the physical distance between the wavelet coefficients. In this case, complete linkage hierarchical clustering is attractive because it yields relatively homogeneous clusters, a key property for subsequent accurate reduction of cluster dimensionality.

Clusterization begins with each $\hat{\mathbf {s}}^{m}$ defining a singleton cluster. At each step, it groups a pair $(A,B)$ of clusters under the condition of minimizing the following distance function:

13 $$\begin{aligned} {\rm{dist}}(A, B) = \max \{\psi (\hat{\mathbf{s}}^{i}, \hat{\mathbf{s}}^{j}): i \in A, j \in B\}, \end{aligned}$$

where

14 $$\begin{aligned} \psi (\hat{\mathbf{s}}^{i}, \hat{\mathbf{s}}^{j}) = \left\{ \begin{array}{cl} 1, \quad {} |\bar{\phi }_{i}-\bar{\phi }_{j}| > \max (2^{l_{i}},2^{l_{j}}) \\ 1-|{\mathrm{cor}}(\hat{\mathbf{s}}^{i}, \hat{\mathbf{s}}^{j})|,\quad {} {\mathrm {otherwise}} , \end{array} \right. \end{aligned}$$

where $\hbox {cor}(\hat{\mathbf {s}}^{i}, \hat{\mathbf {s}}^{j})$ denotes the correlation between $\hat{\mathbf {s}}^{i}$ and $\hat{\mathbf {s}}^{j}, \bar{\phi }_{i} = \int _{\mathbb {R}^{d}} s |\phi _{i}|^{2} ds / \int _{\mathbb {R}^{d}} |\phi _{i}|^{2} ds$ defines de center of mass of $\phi _{i}$ and $l_{i}$ is the scale index of $\phi _{i}$ in the wavelet decomposition. Accordingly, the above dissimilarity measure combines the absolute value of the correlation coefficient and the physical distance between the wavelet coefficients. Clusterization stops when the minimal distance between the clusters is larger than $r$ (i.e. $\min \{ \mathrm{{dist}}(A, B): \forall A, \forall B \} > r$), for some appropriately chosen $r$ thus leading to a list of cluster memberships that characterize the system’s spatial components.

Even though the dissimilarity measure (14) already establishes much of the structure that forms the spatial components of (17), one must decide when to stop clustering by an appropriate value of $r$. Note that the $\hbox {dist}(A, B)$ depends solely on the correlation between the wavelet coefficients in $A$ and $B$. The Fisher z-transform of correlation coefficients, $0.5 \log _{e}(\frac{1+r}{1-r})$, follows a well-known statistic whose upper limit with an $(1-\alpha /2)$ % confidence under the null hypothesis of independence is approximately

15 $$\begin{aligned} u = z_{(1-\alpha /2)}\sqrt{1/(N-3)}, \end{aligned}$$

where $z_{(1-\alpha /2)}$ is the standard normal. Hence, we set the stopping value as

16 $$\begin{aligned} r = 1-|(\exp (2u)+1)/(\exp (2u)-1)| \end{aligned}$$

for $\alpha = 0.05$, which interestingly allows estimating the number of spatial components with reference neither to the actual noise level nor to the number of variables, but solely depending on sample size.

### Within cluster dimensionality reduction

The next step consists of estimating the observation matrix ${\mathbf {A}}$ and system states of (1, 2) by linear dimensional reduction of each spatial cluster identified in the previous step. After clustering the rows of $\hat{\mathbf {S}}$, the $k$-th spatial component ${\mathbf {a}}_{k} {\mathbf {x}}^{k}$ can be approximated by

17 $$\begin{aligned} {\mathbf {Y}}_{k} = \sum _{i \in I_{k}} \phi ^{-1}_{i} \hat{\mathbf {s}}^{i}, \end{aligned}$$

where ${\mathbf {Y}}_{k}$ is an $M \times T$ data matrix, $\phi ^{-1}_{i}$ is the $i$-th column of the inverse of $\varvec{\varPhi }(\varvec{\varPhi }^\mathrm{{T}}$, for wavelet transforms) and $I_{k}$ contains the indexes of the $k$-th cluster. We assume that the rows of ${\mathbf {Y}}_{k}$ have zero mean, otherwise their mean value can be removed after (17).

According to the approximation model,

18 $$\begin{aligned} {\mathbf {Y}}_{k} = {\mathbf {a}}_{k} {\mathbf {x}}^{k} + {\mathbf {E}}_{k}, \end{aligned}$$

where ${\mathbf {E}}_{k}$ is an $M \times T$ approximation error matrix, and one must find ${\mathbf {a}}_{k}$ and ${\mathbf {x}}^{k}$ minimizing the approximation error

19 $$\begin{aligned} \min _{{\mathbf {a}}_{k}, {\mathbf {x}}^{k}} \Vert {\mathbf {Y}}_{k} - {\mathbf {a}}_{k} {\mathbf {x}}^{k}\Vert _{F}, \end{aligned}$$

where $\Vert \cdot \Vert _{F}$ denotes the Frobenius norm.

In fact, each spatial component ${\mathbf {a}}_{k} {\mathbf {x}}^{k}$ is a rank-one $M \times T$ matrix given by the first singular value of ${\mathbf {Y}}_{k}$, i.e.

20 $$\begin{aligned} {\mathbf {Y}}_{k} \approx {\upsigma }_{1} {\mathbf {u}}_{1} {\mathbf {v}}_{1}^{ {T}}, \end{aligned}$$

where $\upsigma _{1}$ is the largest singular value of ${\mathbf {Y}}_{k}$, and where $\mathbf {u}_{1}$ and ${\mathbf {v}}_{1}$ are, respectively, the left-singular vector and the right-singular vectors associated to $\upsigma _{1}$. With no loss of generality, we consider that the norm of ${\mathbf {a}}_{k}$ equals one leading to

21 $$\begin{aligned} {\mathbf {a}}_{k}&= {\mathbf {u}}_{1} \end{aligned}$$

22 $$\begin{aligned} {\mathbf {x}}^{k}&= {\upsigma }_{1} {\mathbf {v}}_{1}^{ {T}}. \end{aligned}$$

### LDSTM parameter estimation

The remainder of the algorithm consists of applying the traditional EM algorithm for ${\mathbf {x}}_{t}$ estimation [27] using the estimators for ${\mathbf {x}}^{k}$ and ${\mathbf {a}}_{k}$ from previous section to set the initial values for ${\mathbf {x}}_{t}$ and ${\mathbf {A}}$. Additionally, during the iterative process, ${\mathbf {A}}$ matrix estimation is modified to accommodate linear equality constraints that ensure well-localized ${\mathbf {a}}_{k}$’s. This is done by solving the following least squares problem:

23 $$\begin{aligned} \min _{{\mathbf {a}}_{k}} \Vert {\mathbf {a}}_{k} {\mathbf {x}}^{k} - {\mathbf {Z}}\Vert ^{2}_{2} \nonumber \\ {\text{subject to}} \; {\mathbf {C}} {\mathbf {a}}_{k} = 0, \end{aligned}$$

where ${\mathbf {C}} = (c_{i,j})_{i,j}$ is an $M \times M$ matrix with $c_{i,i} = 1$ if ${\hbox {VAR}}({\mathbf {s}}^{k}_{i,t}) > 0$ and $c_{i,j} = 0$ otherwise.

## Numerical illustration

**Fig. 2:**
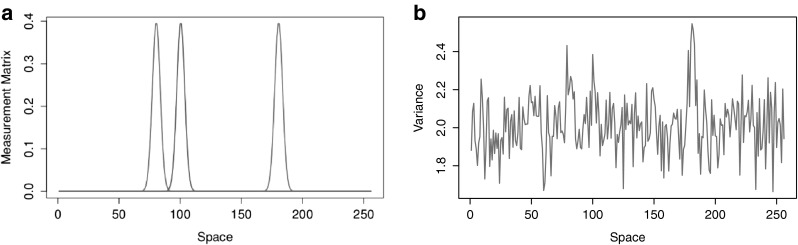
**a** Measurement matrix ${\mathbf {A}}$ and **b** sample variance of the example model with $N=500$ and $\mathrm{{SNR}} = -19$ db

Using simulated data to examine algorithm performance under different conditions, we created a vector time series corresponding to points on a discretized one-dimensional space consisting of $M = 256$ space points whose activity evolves in over a period of $T = 500$ points each. The observation matrix that we used (Fig. 2a) consists of the columns of

$$\begin{aligned} {\mathbf {A}} = [{\mathbf {f}}_{80} {\mathbf {f}}_{180} {\mathbf {f}}_{100}], \end{aligned}$$

where ${\mathbf {f}}_{\mu } = [f_{1,\mu },\ldots ,f_{M,\mu }]^\mathrm{{T}}$ with $f_{i,\mu } = f(i - \mu )$ and $f$ following a discretized Gaussian point-spread function. The observations were corrupted by white Gaussian noise with covariance matrix

$$\begin{aligned} {\mathbf {R}} = \upsigma ^{2} {\mathbf {I}}_{128,128}, \end{aligned}$$

with $\upsigma ^{2}$ accounting for the SNR level defined as $\mathrm{{SNR}} = 10 \log _{10} ({\hbox {VAR}}({\mathbf {s}})/\upsigma ^{2})$ where ${\mathbf {s}} = {\mathrm {vec}}(\left[ {\mathbf {A}} {\mathbf {x}}_{1} \cdots {\mathbf {A}} {\mathbf {x}}_{N} \right] )$. The dynamics of the spatial components evolved according to a first-order autoregressive model $(L=1)$ with

$$\begin{aligned} {\mathbf {H}}_{1} = \left[ \begin{array}{ccc} 0.5 &{} -0.5 &{} 0 \\ 0 &{} 0.5 &{} 0 \\ 0 &{} 0 &{} 0 \end{array} \right] , \end{aligned}$$

and

$$\begin{aligned} {\mathbf {Q}} = \left[ \begin{array}{ccc} 1 &{} 0.5 &{} 0 \\ 0.5 &{} 2 &{} 0 \\ 0 &{} 0 &{} 2 \end{array} \right] . \end{aligned}$$

Figure 2b shows the sample variance for a simulated DSTM using the above parameters under $\mathrm{{SNR}} = -19\hbox {db}$.

**Fig. 3:**
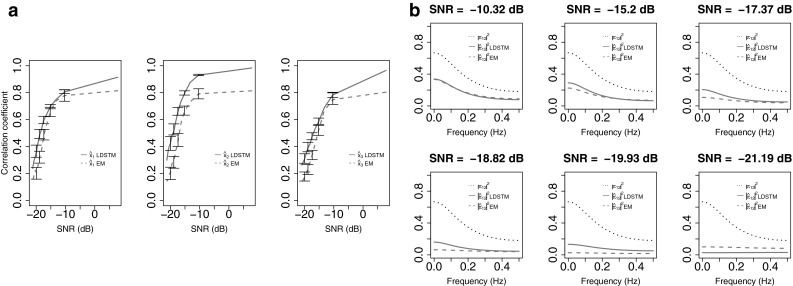
**a** Efficiency comparison between LDSTM (*solid lines*) and EM (*dashed lines*) in recovering source temporal information. *Lines* represent the mean correlation between the simulated hidden state $x_{k,t}$ and the estimated hidden state $\hat{x}_{k,t}$ across $100$ simulations. *Vertical error bars* denote the $95$ % confidence interval of the mean value. **b** *Dotted lines* represent the theoretical PDC of ${\mathbf {x}}_{2}$ towards ${\mathbf {x}}_{1}$ together with estimated PDC values of ${\mathbf {x}}_{2}$ towards ${\mathbf {x}}_{1}$ using LDSTM (*solid*) and EM (*dash*)

We used Daubechies (D2) functions to transform the data and gauged performance by executing 100 Monte Carlo simulations leading to the mean and deviation results as shown in Fig. 3. Algorithm effectiveness was evaluated in terms of how well sources were recovered, as measured by their correlation to the estimated ${\mathbf {x}}_{t}$, and by how well ${\mathbf {H}}_{l}$ and ${\mathbf {Q}}$ could be estimated as evaluated by computing the connectivity between states using Partial Directed Coherence (PDC) [1].

### Simulation results

The mean absolute values of the correlation coefficient between the simulated and estimated sources versus SNR in Fig. 3a show that LDSTM outperforms traditional EM, with very good results for all the three sources even under very unfavourable SNR.

Figure 3b shows PDC from ${\mathbf {x}}_{2}$ towards ${\mathbf {x}}_{1}$ for different SNR levels compared to the corresponding EM estimates. Correct PDC patterns were obtained whose magnitude decreases as SNR decreases but whose overall shape remains.

## Real FMRI data

For further illustration purposes, we used fMRI images from seven healthy volunteers under a resting state protocol (approved by the local ethical committee and under individual informed written consent).

### Image data acquisition

Whole-brain fMRI images ($\hbox {TR}=600\hbox { ms}, \hbox {TE}=33\hbox { ms}, 32$ slices, $\hbox {FOV} = 247 \times 247 \hbox { mm}$, matrix size $128 \times 128$, in-plane resolution $1.975 \times 1.975 \hbox { mm}$, slice thickness $3.5\hbox { mm}$ with $1.8\hbox { mm}$ of gap) were acquired on a 3T Siemens system using a Multiplexed Echo Planar Imaging sequence (multi-band accelerator factor of $4$) [16]. To aid in the localization of functional data, high-resolution T1-weighted images were also acquired with an MPRAGE sequence ($\hbox {TR} = 2500\hbox { ms}, \hbox { TE} = 3.45 \,\hbox {ms}$, inversion time = 1000 ms, $256 \times 256 \hbox { mm}$ FOV, $256 \times 256$ in-plane matrix, $1 \times 1 \times 1 \hbox { mm}$ voxel size, $7\,^{\circ }$ flip angle).

### LDSTM preprocessing

Motion and slice time correction and temporal high-pass filtering (allowing fluctuations above $0.005\,\hbox {Hz}$) were carried out using FEAT $\hbox {v}5.98$. The fMRI data were aligned to the grey matter mask via FreeSurfer’s automatic registration tools (v. 5.0.0) resulting in extracted BOLD signals at regions with preponderantly neuronal cell bodies. To further group analysis by temporal concatenation of the participants’ fMRIs, individual grey matter images were registered to the 3-mm-thick Montreal Neurological Institute (MNI) template using a 12-parameter affine transform. To generate the spatial wavelet transformation, we used 3D Daubechies (D2) functions up to level 3. The model order for the dynamical component in (1) was defined by the Akaike information criterion.

### ICA processing

To compare the LDSTM components with ICA, PICA was performed by multi-session temporal concatenation group ICA (using MELODIC in FSL). Preprocessing included slice time correction, motion correction, skull stripping, spatial smoothing (FWHM equals to 5 mm) and temporal high-pass filtering (allowing fluctuations above $0.005\,\hbox {Hz}$). The functional images were aligned into the standard space by applying 12 degrees-of-freedom linear affine transformation, and its time series were normalized to have variance of unity. The number of components was fixed at 30 to match the distinct pattern of resting state networks (RSN) usually found by other authors [4, 10].

**Fig. 4:**
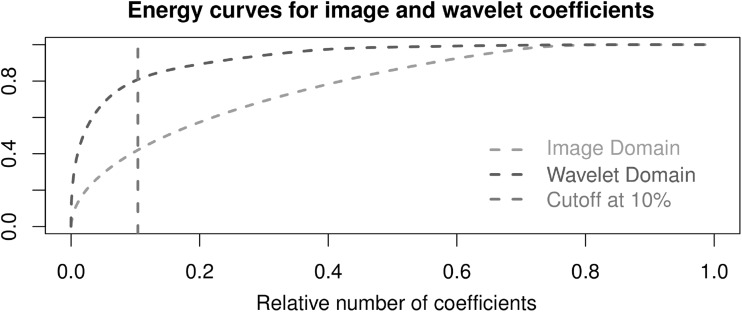
Fraction of cumulative energy in the image (*green*) and wavelet (*red*) domain for the resting state fMRI dataset. The *blue vertical line* crosses the fraction of cumulative energy represented by 10 % of the most energetic coefficients in the image (40 %) and wavelet (80 %) domains

### Image results

#### LDSTM results

Figure 4 illustrates the advantage of wavelet transforming resting state fMRI datasets: the entropy in the image domain is much larger than that in the wavelet domain. This means that only a few wavelet coefficients are enough to account for much of the signal energy. In the example, 10 % of the wavelet coefficients explain 80 % of the image energy which is two times more than the 10 % of the most powerful image domain coefficients which represent just 40 %.

**Fig. 5:**
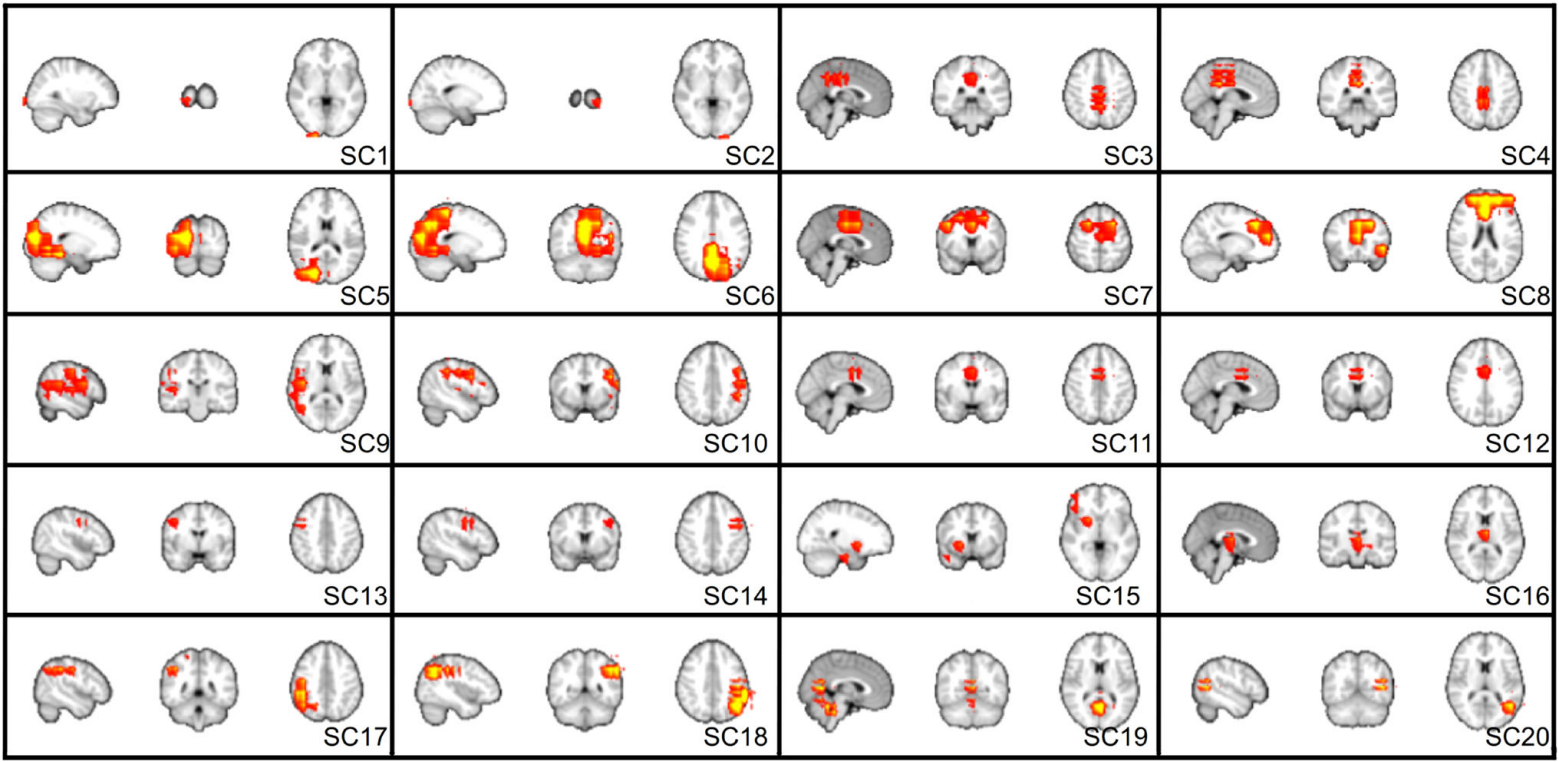
Cortical and subcortical components identified by LDSTM

**Fig. 6:**
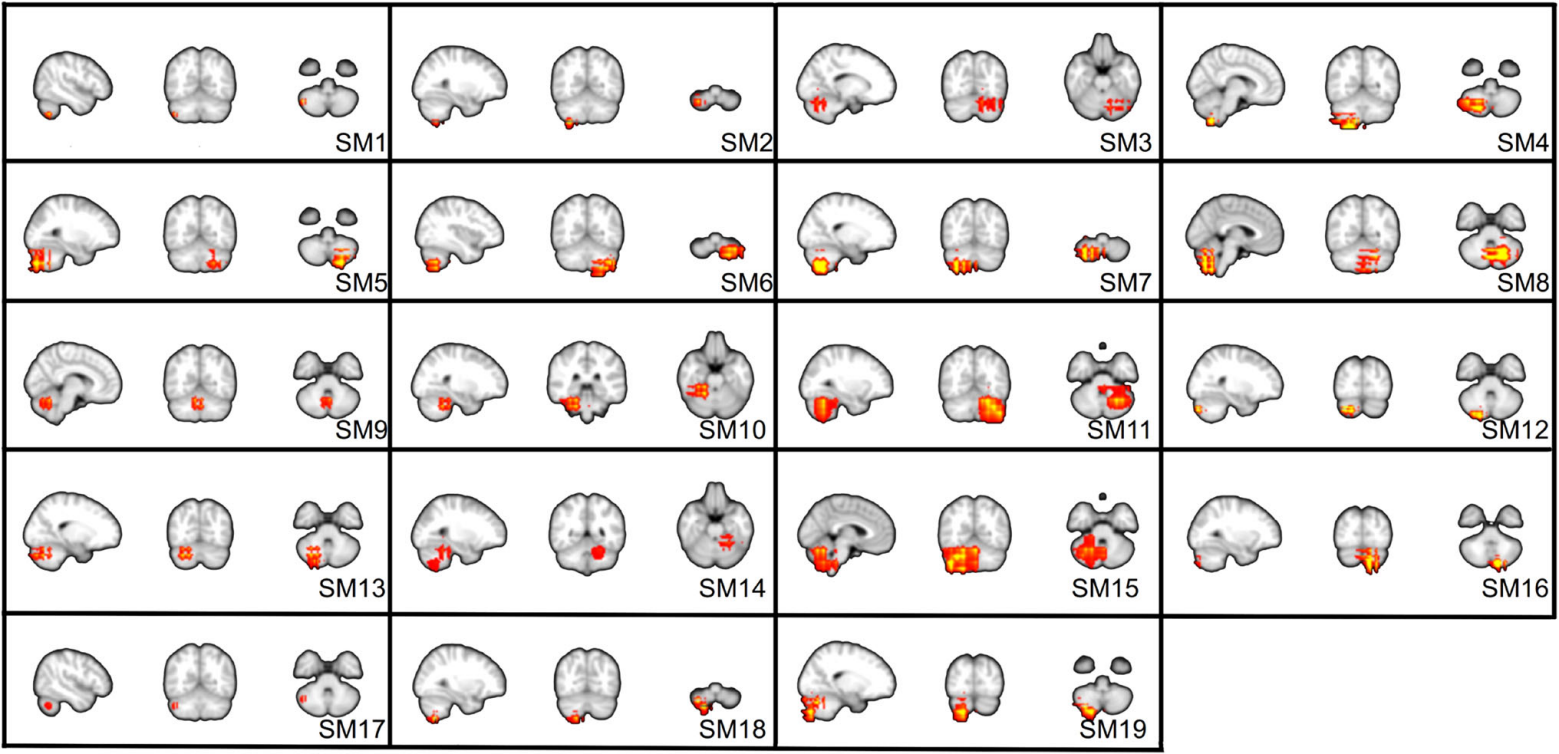
Cerebellum components identified by LDSTM

LDSTM analysis identified thirty nine well-localized spatial components comprising cortical (18), subcortical (2) and cerebellar (19) regions. Cortical and subcortical spatial components (${\mathbf {a}}_{k}$’s) are shown in Fig. 5 which includes the following anatomical areas: occipital cortex ($\hbox {SC}1$ and $\hbox {SC}2$), lateral and superior occipital gyrus ($\hbox {SC}5, \hbox {SC}6$ and $\hbox {SC}20$), superior temporal gyrus ($\hbox {SC}9$ and $\hbox {SC}10$), precentral gyrus ($\hbox {SC}13$ and $\hbox {SC}14$), superior parietal gyrus ($\hbox {SC}17$ and $\hbox {SC}18$), precuneus ($\hbox {SC}3$ and $\hbox {SC}19$) and posterior cingulate ($\hbox {SC}4$), inferior frontal gyrus and anterior cingulate ($\hbox {SC}7, \hbox {SC}8, \hbox {SC}11$ and $\hbox {SC}11$)) and thalamus ($\hbox {SC}15$ and $\hbox {SC}16$). Cerebellar regions also form well-localized bilateral activity patterns as shown in Fig. 6.

**Fig. 7:**
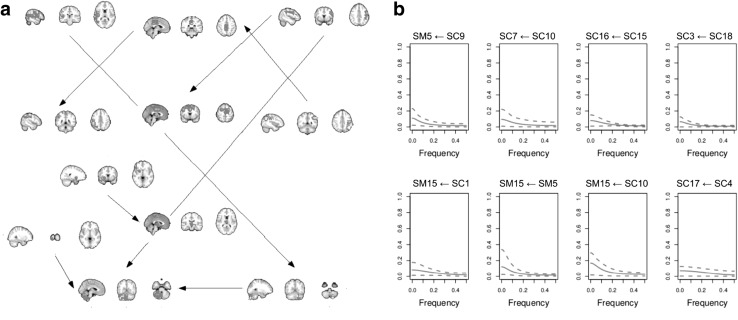
FMRI resting state analysis using LDSTM. *Numbers* represent different components. Components numbered twice represent two components located at the same region. **a** Connectivity map showing components whose system states are connected via the PDC. **b** PDC plots for each arrow drawing in **a** *Dashed lines* denote the $95$ % confidence interval of the mean value (*solid lines*)

The absence of artificial stochastic model constraints permitted exposing the dynamic connectivity between the identified components. Figure 7a summarizes the connectivity network estimated using PDC applied to the reconstructed system components. In addition, PDC also highlights that resting state connectivity is present mainly at low frequencies (Fig. 7b), corroborating several studies of resting state brain connectivity [4].

#### ICA results

**Fig. 8:**
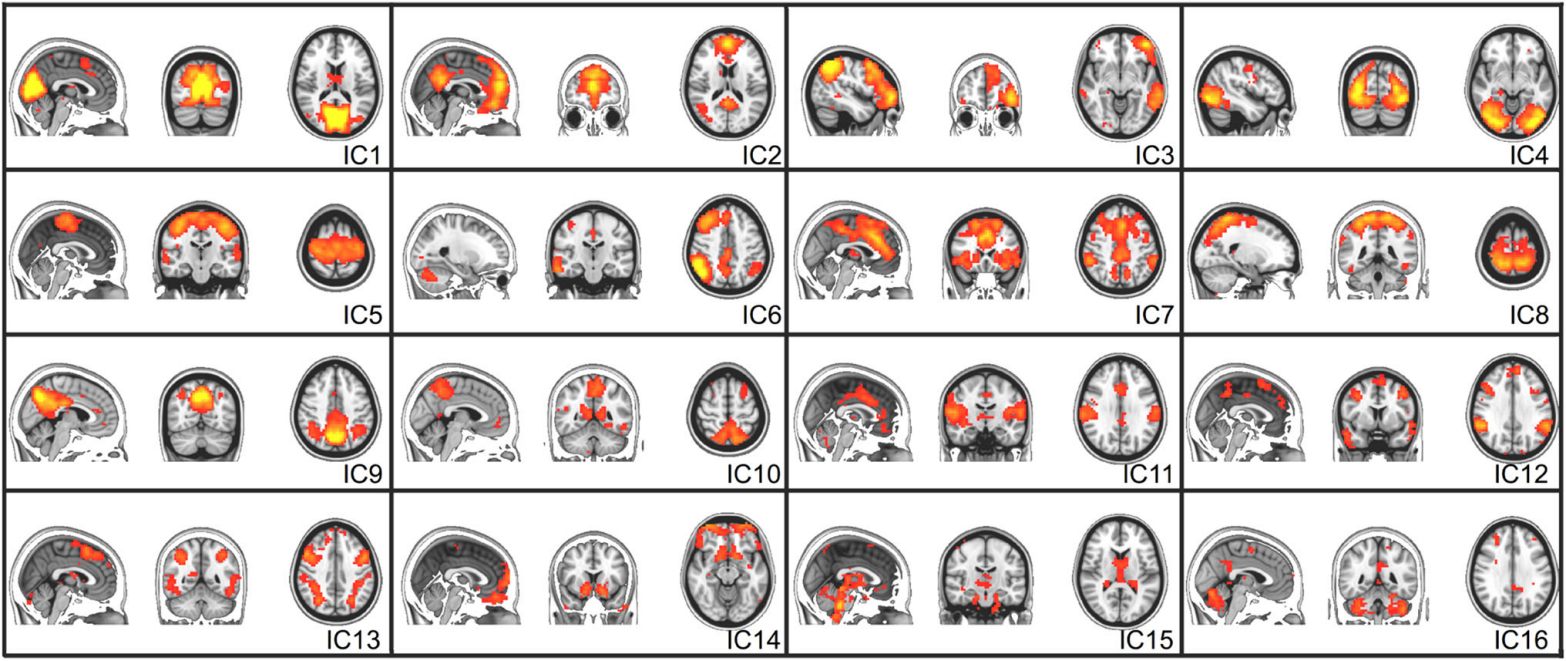
ICA spatial components. The components are sorted according to their relative percentage of variance from *top left* to *bottom right*

Among the 30 component maps obtained by performing a PICA across all participants, 14 components were considered artifactual components due to scanner and physiological noise. Their signal variances are related to cerebrospinal fluid and white matter, head motion and large vessels. Figure 8 depicts fourteen functional components related to previously report resting state studies. They comprise the default mode network (IC$2$, IC$9$, IC$10$) and brain regions involved in visual (IC$1$, IC$4$), auditory/motor (IC$5$), sensory/motor (IC$8$), attentional (IC$7$, IC$6$, IC$12$, IC$13$) and executive functions (IC$7$, IC$11$, IC$14$). In addition, we found 2 components rarely reported in resting state studies. One is a cerebellum component (IC$16$) and the other is a brainstem component (IC$15$).

## Discussion

Local dimension-reduced modelling (LDSTM) as presented here addresses an approach to source estimation and localization in resting state fMRI data analysis that dispenses with artificial stochastic model assumptions, such as those used in classical blind source separation (principal component analysis (PCA), independent component analysis (ICA) and non-negative matrix factorization (NMF) [3, 18, 19, 21]). In addition to being sparse, the columns of the observation matrix act as point-spreading functions that allow system sources and their observation matrix to be identified via LSCA [32] of the whole fMRI dataset.

The cortical components identified by LDSTM (Fig. 5) reflect most of the data variability and coincide with traditional resting state regions observed across different individuals, data acquisition and analysis techniques. They comprise the default mode network $(\hbox {SC}8)$ and brain regions involved in visual $(\hbox {SC}1, \hbox {SC}2, \hbox {SC}5, \hbox {SC}6)$, motor $(\hbox {SC}13, \hbox {SC}14, \hbox {SC}7)$ and attentional functions $(\hbox {SC}9, \hbox {SC}10, \hbox {SC}17, \hbox {SC}18)$, indicating that most of the ICA components (Fig. 8) can in fact be decomposed into several local sparse components. However, the present results draw attention to the fact that they were obtained without any additional assumption, such as source independence and/or stationarity. All that was assumed was ${\mathbf {a}}_{k}$ spatial localization, which goes along the line of [11]’s observation that ICA effectiveness for brain FMRI is linked to their ability to handle sparse sources rather than independent ones. This could be explained by pointing out that ICA preprocessing steps involve projecting the data into a reduced-dimensional subspace via the singular value decomposition which in turn confines the sources to regions of high signal variance.

PDC analysis shows a network where the information flows from regions in the superior parietal cortex (SPC) to regions in the cerebellum (CER) and anterior cingulate. As expected, the right SPC sends information to the left CER, and left SPC sends information to the right CER. Although the relationship between these structures is known, this stresses two main systems engaging in the mentioned network. The connectivity between SPC and CER is in line with recent studies showing evidence of a cerebellar-parietal network involved in phonological storage [22]. In addition, visual–parietal–cerebellar interactions are expected by following studies of effective connectivity using FMRI [20]. We also observe a network running from the left to right parietal cortex passing through the posterior cingulate. Altogether, we believe that our results provide insight into the mechanisms of how the regions of the fronto-parietal network interact. It also highlights understudied aspects of the cerebellum in this network during resting state.

In our model, LDSTM identified approximately 50 % of components in the cerebellum. This result is surprising as the rate of cerebellar components identified in resting state using ICA is below 20 % in general [4]. Some of these regions seem to be related to noise sources for being located near cerebellar arteries and veins. The components SM1, SM2, SM12, SM17 and SM18 run in the superior surface of the cerebellum near to the superior cerebellar veins, while the components SM8 and SM9 extend into the end of the straight sinus near to internal cerebral veins. On the other hand, the idea that the cerebellum should present as many components as the cortex is encouraging. Many recent fMRI studies have shown that different cerebellar regions are critical for processing higher-order functions in different cognitive domains, in the same way as it occurs in the cortex [30]. In these studies, it is worth mentioning that cerebellar clusters are always smaller than those of corresponding functionality in the cortex. We believe that some differences between ICA and LDSTM may be explained in part by the features along the domain in which they represent the sources.

Since spatial wavelet analysis efficiently encodes the data neighbourhood information via a orthogonal transformation, the present method properly addresses a number of issues involving whole-brain connectivity estimation. The first one is associated to the lack of knowledge about the spatial localization of the sources. The method provides a data-driven approach to locate the main sources of data variability, thus avoiding the effects and uncertainties due to a priori region of interest delineation. The second aspect is that the new method naturally employs multi-scale transformations to create a compact model of the images, a feature of growing importance as higher-resolution images are sure to become available in the near future and whose computational processing load may be thereby substantially mitigated. Finally and most importantly, unlike ICA, the method permits deeper connectivity analysis between the identified spatial components as no independence assumption is made ’a priori’.

Various method extensions are possible, especially when it comes to estimate appropriate regularization parameter choice as a function of the amount of noise present in the data. In the present implementation, spatial noise is assumed homogeneous and normally distributed which implies a chi-squared distribution for wavelet coefficient variance. Examination of wavelet coefficients variance for real fMRI data, however, points to the need to consider heavy-tailed distributions, so that a more general approach is currently being developed to estimate wavelet domain noise variance from a finite mixture of exponential distributions that could then be used to quantify the level of data sparsity.

## Conclusions

Here, an EM-based algorithm was presented for LDSTM identification. By projecting high-dimensional datasets into smoothness spaces, one can describe the system’s spatial components via a reduced number of parameters. Further dimension reduction and denoising is obtained by soft-vector thresholding under contiguity-constrained hierarchical clustering. Finally, simulated results corroborate that the new algorithm can outperform the traditional EM approach even under mild conditions. Even with very large datasets as in the fMRI example, LDSTM shows promise in its ability to parcelate the human brain into well-localized physiologically plausible regions of spatio-temporal brain activation patterns.

## References

[1] Baccala LA, de Brito CSN, Takahashi DY, Sameshima K (2013). Unified asymptotic theory for all partial directed coherence forms. Philos Trans R Soc A.

[2] Bach F, Jenatton R, Mairal J, Obozinski G (2011) Structured sparsity through convex optimization. arXiv e-print arXiv:1109.2397

[3] Beckmann CF, Smith SM (2004). Probabilistic independent component analysis for functional magnetic resonance imaging. IEEE Trans Med Imaging.

[4] Biswal BB, Mennes M, Zuo X-N, Gohel S, Kelly C, Smith SM, Beckmann CF, Adelstein JS, Buckner RL, Colcombe S, Dogonowski A-M, Ernst M, Fair D, Hampson M, Hoptman MJ, Hyde JS, Kiviniemi VJ, Ktter R, Li S-J, Lin C-P, Lowe MJ, Mackay C, Madden DJ, Madsen KH, Margulies DS, Mayberg HS, McMahon K, Monk CS, Mostofsky SH, Nagel BJ, Pekar JJ, Peltier SJ, Petersen SE, Riedl V, Rombouts SARB, Rypma B, Schlaggar BL, Schmidt S, Seidler RD, Siegle GJ, Sorg C, Teng G-J, Veijola J, Villringer A, Walter M, Wang L, Weng X-C, Whitfield-Gabrieli S, Williamson P, Windischberger C, Zang Y-F, Zhang H-Y, Castellanos FX, Milham MP (2010). Toward discovery science of human brain function. Proc Natl Acad Sci USA.

[5] Blumensath T, Jbabdi S, Glasser MF, Van Essen DC, Ugurbil K, Behrens TEJ, Smith SM (2013). Spatially constrained hierarchical parcellation of the brain with resting-state fMRI. NeuroImage.

[6] Chen SS, Donoho DL, Saunders MA (1998). Atomic decomposition by basis pursuit. SIAM J Sci Comput.

[7] Combettes PL, Wajs VR (2005). Signal recovery by proximal forward-backward splitting. Multiscale Model Simul.

[8] Cortes J (2009). Distributed kriged kalman filter for spatial estimation. IEEE Trans Autom Control.

[9] Cressie N, Wikle CK (2011). Statistics for spatio-temporal data.

[10] Damoiseaux JS, Rombouts SARB, Barkhof F, Scheltens P, Stam CJ, Smith SM, Beckmann CF (2006). Consistent resting-state networks across healthy subjects. Proc Natl Acad Sci.

[11] Daubechies I, Roussos E, Takerkart S, Benharrosh M, Golden C, D’Ardenne K, Richter W, Cohen JD, Haxby J (2009). Independent component analysis for brain fMRI does not select for independence. Proc Natl Acad Sci USA.

[12] Daubechies I, Defrise M, De Mol C (2003) An iterative thresholding algorithm for linear inverse problems with a sparsity constraint. arXiv e-print math/0307152

[13] Dempster AP, Laird NM, Rubin DB (1977). Maximum likelihood from incomplete data via the EM algorithm. J R Stat Soc Ser B.

[14] Dewar M, Scerri K, Kadirkamanathan V (2009). Data-driven spatio-temporal modeling using the integro-difference equation. IEEE Trans Signal Process.

[15] Donoho DL, Johnstone IM, Kerkyacharian G, Picard D (1995). Wavelet shrinkage: asymptopia?. J R Stat Soc Ser B (Methodol).

[16] Feinberg DA, Moeller S, Smith SM, Auerbach E, Ramanna S, Glasser MF, Miller KL, Ugurbil K, Yacoub E (2010). Multiplexed echo planar imaging for sub-second whole brain FMRI and fast diffusion imaging. PLoS One.

[17] Figueiredo MAT, Nowak RD (2003). An EM algorithm for wavelet-based image restoration. IEEE Trans Image Process.

[18] Friston KJ, Frith CD, Liddle PF, Frackowiak RSJ (1993). Functional connectivity: the principal-component analysis of large (PET) data sets. J Cereb Blood Flow Metab.

[19] Georgiev P, Theis F, Cichocki A, Bakardjian H, Pardalos PM, Boginski VL, Vazacopoulos A (2007). Sparse component analysis: a new tool for data mining. Data mining in biomedicine, number 7 in Springer optimization and its applications.

[20] Kellermann T, Regenbogen C, De Vos M, Mnang C, Finkelmeyer A, Habel U (2012). Effective connectivity of the human cerebellum during visual attention. J Neurosci.

[21] Lohmann G, Volz KG, Ullsperger M (2007). Using non-negative matrix factorization for single-trial analysis of fMRI data. Neuroimage.

[22] Macher K, Bhringer A, Villringer A, Pleger B (2014). Cerebellar-parietal connections underpin phonological storage. J Neurosci.

[23] Mallat SG (2009). A wavelet tour of signal processing the sparse way.

[24] Mardia KV, Goodall C, Redfern EJ, Alonso FJ (1998). The kriged kalman filter. Test.

[25] Rauch HE, Striebel CT, Tung F (1965). Maximum likelihood estimates of linear dynamic systems. J Am Inst Aeronaut Astronaut.

[26] Scerri K, Dewar M, Kadirkamanathan V (2009). Estimation and model selection for an IDE-based spatio-temporal model. IEEE Trans Signal Process.

[27] Shumway RH, Stoffer DS (1982). An approach to time series smoothing and forecasting using the em algorithm. J Time Ser Anal.

[28] Stoodley CJ, Schmahmann JD (2009). Functional topography in the human cerebellum: a meta-analysis of neuroimaging studies. Neuroimage.

[29] Theophilides CN, Ahearn SC, Grady S, Merlino M (2003). Identifying west nile virus risk areas: the dynamic continuous-area space-time system. Am J Epidemiol.

[30] Tibshirani R (1994). Regression shrinkage and selection via the lasso. J R Stat Soc Ser B.

[31] Vieira G, Amaro E, Baccala LA, Hutchison D, Kanade T, Kittler J, Kleinberg JM, Kobsa A, Mattern F, Mitchell JC, Naor M, Nierstrasz O, Pandu Rangan C, Steffen B, Terzopoulos D, Tygar D, Weikum G, lezak D, Tan A-H, Peters JF, Schwabe L (2014). Local dimension-reduced dynamical spatio-temporal models for resting state network estimation. Brain informatics and health.

[32] Vieira G, Amaro E, Baccala LA (2014) Local sparse component analysis for blind source separation: an application to resting state fmri. In: Proceedings of IEEE EMBS conference, IEEE 10.1109/EMBC.2014.6944899 25571267

[33] Wikle CK, Cressie N (1999). A dimension-reduced approach to space-time kalman filtering. Biometrika.

[34] Woolrich MW, Jenkinson M, Michael Brady J, Smith SM (2004). Fully bayesian spatio-temporal modeling of FMRI data. IEEE Trans Med Imaging.

[35] Wright SJ, Nowak RD, Figueiredo MAT (2009). Sparse reconstruction by separable approximation. IEEE Trans Signal Process.

[36] Yuan M, Lin Y (2006). Model selection and estimation in regression with grouped variables. J R Stat Soc.

[37] Zalesky A, Fornito A, Harding IH, Cocchi L, Ycel M, Pantelis C, Bullmore ET (2010). Whole-brain anatomical networks: Does the choice of nodes matter?. NeuroImage.

[38] Zhao P, Rocha G (2009) The composite absolute penalties family for grouped and hierarchical variable selection. Ann Stat 37(6A):3468–3497 arXiv e-print arXiv:0909.0411

[39] Zibulevsky M, Pearlmutter BA (2001). Blind source separation by sparse decomposition in a signal dictionary. Neural Comput.

